# The impact of the COVID-19 pandemic on the diet, training habits and fitness of Masters cyclists

**DOI:** 10.1177/02601060211002350

**Published:** 2022-12

**Authors:** Keely A Shaw, Leandy Bertrand, Dalton Deprez, Jongbum Ko, Gordon A Zello, Philip D Chilibeck

**Affiliations:** 1 College of Kinesiology, 7235 University of Saskatchewan, Canada; 2 College of Pharmacy and Nutrition, 7235 University of Saskatchewan, Canada

**Keywords:** Cycling, exercise, nutrition, training stress score, sedentary behavior

## Abstract

**Background:** The number of Masters-level athletes (≥ 35 years of age) taking part in cycling has increased in the past years which may have beneficial effects on their health. The restrictions brought on by the COVID-19 pandemic has the potential to negatively impact the diet, training and fitness of these individuals due to restrictions in place to slow the spread of the virus. **Aim:** To investigate how the COVID-19 pandemic impacted the diet, training and fitness of Masters-level cyclists. **Methods:** 32 Masters cyclists (12 males, 20 females; mean age 47 ± 10 years) completed two incremental exercise tests one month apart during the pandemic to assess sport-specific fitness. Participants also completed online questionnaires to report their sedentary behavior and dietary intake before and during the pandemic, and their training volume and intensity for a specified week in February (before the pandemic) and each of March, April and May (during the pandemic). **Results:** No differences were seen in fitness (*p* = 0.6), training volume (*p* = 0.24) or intensity (*p* = 0.79) and sedentary behavior (*p* = 0.14) during the pandemic. Energy intake was unchanged (*p* = 1.0) during the pandemic, but participants consumed lower amounts of key nutrients such as fiber, vitamin A, omega-3 fatty acids and potassium (*p* < 0.05) while consuming more alcohol (*p* = 0.008) and vitamin C (*p* = 0.03). **Conclusions:** Our data shows that the COVID-19 pandemic has undesirable effects on nutrient and alcohol intake of Masters cyclists without impacting their training regimes, which may have adverse effects on their overall health and fitness in the long term.

## Introduction

Participation in physical activity has various physical and cognitive benefits as individuals age (Cavanagh et al., 1998; Maula et al., 2019). Despite a global decrease in recreational physical activity among the aging population, participation in competitive sport amongst this age group (termed Masters athletes) has increased substantially in recent years (Baker et al., 2009; Bradley et al., 2005). The sport of cycling in particular has seen considerable growth in the Master population with over half of USA Cycling license holders falling into the Masters age category in 2014 (Appleby and Dieffenbach, 2016).

Following the declaration of the COVID-19 global pandemic by the World Health Organization, global leaders began to impose various restrictions to prevent spread of COVID-19. Due to the presumed asymptomatic carrier transmission of the virus (Bai et al., 2020), strategies for containing the virus often included various degrees of ‘lock-down’ worldwide. Some countries opted to close non-essential services and enforce social distancing policies (Greenstone and Nigam, 2020), with others enforcing rigorous compliance to home quarantine (Lau et al., 2020). This resulted in closures to gyms and other limitations for participating in physical activity since individuals were encouraged not to leave their home except for when absolutely necessary (Lau et al., 2020). In addition to the impact on physical activity, the ability to access and prepare healthy meals that meet nutritional needs may be comprised and accentuated in older adults.

Older adults may be at increased risk for severe COVID-19 related illness and death compared to their younger counterparts (CDC COVID-19 Response Team, 2020). Therefore, older adults have likely spent more time at home than other age groups. Closures to fitness facilities and limitations to outdoor recreational activities put older adults at increased risk for negative health and fitness outcomes related to the COVID-19 pandemic. Nutrition has a critical role in the optimal functioning of the immune system and the resistance to infection, especially nutrients such as vitamin B6, vitamin C and zinc, among others (Butler and Barrientosa, 2020; Lange and Nakamura, 2020; Wintergerst et al., 2007). Further, poor nutrition increases the risk of some health conditions such as diabetes and cardiovascular disease, which may lead to further serious health consequences if COVID-19 is contracted (McRae, 2017; Nishiga et al., 2020). While the term ‘older adult’ generally applies to individuals older than 65 years of age, athletes may be classified as ‘Masters athletes’ at various age points depending on the sport, with those 18 years and older being considered a Master in swimming, 30 years in cycling, 35 years in athletics (i.e. track and field) and 50 years in golf (Concannon et al., 2012). High levels of cardiorespiratory fitness may offer some protective effects against the COVID-19 virus through reducing early amplified proinflammatory responses in patients infected (Jiménez-Pavón et al., 2020). Maintaining high levels of cardiovascular fitness may, therefore, be important for protection in older adults.

The purpose of the current study was to investigate the impact of the COVID-19 pandemic on the training and fitness levels, as well as diet quality, of Masters-level cyclists.

## Methods

### Participants

Masters cyclists – defined as a cyclist of 35 years or older for track cyclists or 30 years and older for all other disciplines, and who engages regularly in local club rides or competes at the provincial, regional, national and international age-group competition (Appleby and Dieffenbach, 2016) – were recruited in June and July of 2020 via social media (shared posters on Facebook and Twitter, direct message on Facebook) to take part in the research study. Participants were deemed eligible if they cycled regularly (more than 1 h, three times per week) and were 35 years and older for track cyclists or 30 years and older for all other disciplines. Individuals who were not cycling regularly for reasons other than the COVID-19 pandemic (e.g. injury) or were not proficient in English were excluded. Informed consent was implied by clicking ‘next’ after reading the informed approved consent form that appeared on the first page of the first questionnaire. Ethical approval to conduct the study was obtained from the University of Saskatchewan Research Ethics Board.

### Study design

Questionnaires were distributed to participants via email throughout the months of June and July 2020 and responses were collected using the online platform Survey Monkey (San Mateo, USA). Upon enrollment, participants were provided links for two surveys: the first contained the informed consent document, demographic information and a food frequency questionnaire (FFQ) used to assess dietary intake, adapted for use in the Canadian population (Csizmadi et al., 2007). This questionnaire presents a variety of foods and participants report how often they eat that food as well as how much they consumed each time. Participants were instructed to complete this questionnaire in relation to their typical diet prior to the COVID-19 pandemic (i.e. before March 2020). This questionnaire also asked participants to report, on average, how much sedentary screen time they engaged in per day prior to the pandemic. The second link contained a questionnaire where participants were asked to report their training volume (total training time in the specified week) and intensity (quantified as Training Stress Score, TSS), a measure automatically calculated by their cycling computer based on heart rate or power output and duration (for more information on the calculation of TSS see van Erp et al. 2019) for a specified week in each of February (before the pandemic), March, April and May (during the pandemic). A minimum of a week later, participants were provided a link to a questionnaire that contained the same FFQ and sedentary behavior questions on the first questionnaire and they were instructed to complete it with regards to their typical behaviors and dietary intake during the pandemic (i.e. after March 2020). Participants also completed two sport-specific fitness tests separated by one month. The first was completed upon enrollment in the study and the second a month later. One month between tests was chosen as Madsen et al. (1993) have found this time period to be sufficient to decrease endurance capacity in times of detraining. Fitness tests took place in the participant’s own home and were self-administered (to maintain social distancing) using their own equipment (i.e. bike, heart-rate monitor, power meter, cycling computer). The protocol for the fitness test included a 5-min self-selected warmup, after which power output was increased by 10 watts every minute, starting at 100 watts, until participants could no longer manage the output (volitional fatigue). Heart rate, power output and test duration were captured by the individual’s cycling computer and emailed to researchers for analysis. As participants used their own equipment, equipment used varied. However, the power meters and heart-rate monitors commonly used have good validity, as indicated by strong correlation with the gold standard (*r* ≥ 0.90), reported by Bouillod et al. (2017) and Terbizan et al. (2002), respectively.

Dietary data were analyzed using the Food Processor Software (ESHA Research, Version 11.1, Salem, Orlando, USA), which produced an estimate of daily energy, micronutrient and macronutrient intake for each time period (before and during the pandemic). Fitness test information was assessed using Garmin Connect (Olathe, USA).

### Statistics

Data were analyzed using JASP statistical software, version 0.10.2 (2013-2019, University of Amsterdam). Data were assessed for normality through observation of skewness and kurtosis. Fitness data (test duration, heart rate and power output) were analyzed using a 2 × 2 factorial ANOVA (sex × time). Training metrics were assessed using a 2 × 4 factorial ANOVA (sex × month) and any observed differences were further analyzed using a Bonferroni *post-hoc* analysis. Training volume and intensity were further assessed using a dependent *t*-test to assess February (before the pandemic) to the average of March, April and May (during the pandemic) as well as a comparison between February and May (when motivation may be the lowest). Sedentary time before and during the COVID-19 pandemic was assessed using a paired-samples (dependent) *t*-test. Dietary data violated assumptions of normality and therefore were assessed using non-parametrical statistics. Differences between time points (before and during the pandemic) were assessed using the Friedman Test, while differences between males and females were assessed using the Kruskal–Wallis Test. In the case of a significant main effect for time, a Friedman test was run on male and female data separately to assess if one sex or the other contributed more to the difference. Significance was accepted at *p* < 0.05. Data are presented as mean ± SD.

## Results

Thirty-nine Masters-level cyclists (22 females, 17 males; age 46 ±10 years) provided informed consent. However, seven (five males, two females) failed to complete the follow-up measures and, therefore, were removed from analysis. The training habits of the current sample are in line with the categorization of ‘recreational cyclists’, as described by Priego Quesada and colleagues (2018).

### Training metrics

Training metric data are displayed in Table 1. No effect of an interaction was observed for training volume (*p* = 0.24), nor for main effects of sex (*p* = 0.82) or time (*p* = 0.81). A comparison of February data and the average of March, April and May also produced no significant differences (*p* = 0.96). No significant differences were observed when February data were compared to May (*p* = 0.44). No interaction effect was observed for intensity (*p* = 0.79). There were no main effects for either sex (*p* = 0.46) or time (*p* = 0.09). When February data was compared to the average of March, April and May, no differences were observed (*p* = 0.60). Similarly, when February was compared to May, no differences were found (*p* = 0.44).

**Table 1.** Training metrics.

	Mean volume ± SD (min)	Mean intensity ± SD
February	637 ± 326	578 ± 292
March	634 ± 317	558 ± 224
April	657 ± 308	573 ± 189
May	574 ± 291	512 ± 171

### Fitness

No significant interaction between time and sex were observed for test duration (*p* = 0.60), average power output (*p* = 0.99), maximum power output (*p* = 0.21), average heart rate (*p* = 0.95), nor maximum heart rate ( *p* = 0.94). No effects of time were observed for any of the measured outcomes (*p* > 0.05). As would be expected, an effect of sex was observed for maximum and average power. There was also an effect of sex for maximum and average heart rate. Females had a significantly lower maximum (*p* < 0.001) and average power output (*p* < 0.001) and had a significantly higher maximum (*p* = 0.03) and average (*p* = 0.009) heart rate. Data are displayed in Table 2.

**Table 2.** Heart rate, power output and test duration.

	Test duration (min)		Average power (W)		Maximum power (W)		Average heart rate (bpm)		Maximum heart rate (bpm)	
	Pre	Post	Pre	Post	Pre	Post	Pre	Post	Pre	Post
Male	30.1 ± 6.8	29.1 ± 8.2	237 ± 33	238 ± 37	378 ± 51	422 ± 60	125 ± 12	126 ± 12	167 ± 13	169 ± 17
Female	24.3 ± 9.2	26.4 ± 7.4	176 ± 42	178 ± 41	317 ± 62	308 ± 48	136 ± 8	137 ± 10	179 ± 12	180 ± 14

Data are presented as mean ± SD.

Main effect of sex for average power (*p* < 0.001), max power (*p* < 0.001), average heart rate (*p* = 0.009) and max heart rate (*p* = 0.025).

bpm, beats per minute.

### Sedentary behavior

No significant differences were observed for time engaged in sedentary behavior before versus during the pandemic (6.15 ± 2.6 v. 6.73 ± 2.5 h; *p* = 0.14).

### Dietary intake

Dietary intake data are shown in Table 3. A main effect of time was observed for total fiber (*p* = 0.03), vitamin A (*p* = 0.01), vitamin C (*p* = 0.03), folate (*p* = 0.01), potassium (*p* = 0.01), omega-3 fatty acids (*p* = 0.01) and alcohol (*p* = 0.01). Intakes of vitamin A, potassium, omega-3 and fiber decreased during compared to before the pandemic while vitamin C and alcohol increased during compared to before the pandemic. Decreases in fiber (*p* = 0.01), vitamin A (*p* = 0.01), folate (*p* = 0.01), potassium (*p* = 0.003) and omega-3 (*p* = 0.04) were driven by females, while increased intakes of vitamin C were driven by males (*p* = 0.04) during the pandemic. Time differences in alcohol does not appear to be due to sex. A difference between males and females was observed for energy (*p* < 0.001), protein (*p* < 0.001), carbohydrate (*p* = 0.002), fiber (*p* = 0.02), sugar (*p* = 0.003), fat (*p* < 0.001), saturated fat (*p* < 0.001), cholesterol (*p* = 0.02), vitamin K (*p* = 0.003), potassium (*p* = 0.02), sodium (*p* = 0.03), omega-3 (*p* < 0.011) and caffeine (*p* = 0.007), with females consuming lower amounts of all, except for vitamin K and caffeine.

**Table 3.** Dietary intake before and during the COVID-19 pandemic.

Nutrient	Before pandemic		During pandemic		*p*-value for time	*p*-value for sex
	Males	Females	Males	Females		
Energy (kcal)	2803 ± 654	2156 ± 576	2990 ± 736	1952 ± 472	1	0.001*
Carbohydrate (g)	350 ± 91	292 ± 94.6	359.6 ± 98	257 ± 77	0.715	0.002*
Fiber (g)	43 ± 11	39 ± 20	40 ± 9	32 ± 15	0.028*	0.022*
Protein (g)	131 ± 51	104 ± 31	131 ± 29	92 ± 23	0.273	< 0.001*
Fat (g)	105 ± 35	68 ± 22	110 ± 45	62 ± 17	0.273	< 0.001*
Saturated fat (g)	27.4 ± 8.0	18.1 ± 7.1	27.4 ± 7.9	16.4 ± 6.3	0.715	< 0.001*
Omega-3 (g)	3.4 ± 2.1	2.0 ± 0.8	2.9 ± 1.7	1.9 ± 0.9	0.011*	< 0.001*
Cholesterol (mg)	437 ± 225	283 ± 149	447 ± 253	283 ± 149	0.715	0.015*
Sugar (g)	149 ± 57	127 ± 60	148 ± 45	102 ± 35	0.465	0.003*
Vitamin A (IU)	5417 ± 4261	5996 ± 4581	3662 ± 764	3900 ± 3264	0.011*	0.86
Vitamin B1 (mg)	2.7 ± 1.2	2.6 ± 1.4	2.7 ± 1.3	2.3 ± 1.3	0.273	0.362
Vitamin B2 (mg)	3.8 ± 2.0	4.1 ± 2.2	4.1 ± 1.6	3.5 ± 1.8	0.715	0.517
Vitamin B3 (mg)	41 ± 17	35 ± 13	40 ± 13	30 ± 11	0.465	0.068
Vitamin B6 (mg)	4.35 ± 2.0	4.3 ± 2.1	4.2 ± 1.6	3.5 ± 1.8	0.144	0.402
Vitamin B12 (µg)	8.3 ± 6.0	9.3 ± 7.4	9.1 ± 5.6	7.3 ± 5.9	0.715	0.303
Vitamin C (mg)	268 ± 148	354 ± 296	220 ± 113	416 ± 424	0.028*	0.162
Vitamin D (IU)	601 ± 505	824 ± 584	637 ± 563	791 ± 582	0.715	0.427
Vitamin E mg)	48 ± 79	24 ± 12	26 ± 11	47 ± 120	0.715	0.228
Folate (µg)	704 ± 247	813 ± 401	711 ± 279	731 ± 416	0.011*	0.871
Vitamin K (µg)	371 ± 129	1042 ± 981	327 ± 179	652 ± 529	0.144	0.003*
Calcium (mg)	1539 ± 778	1815 ± 862	1793 ± 539	1511 ± 702	0.465	0.769
Iron (mg)	24 ± 7	33 ± 19	25 ± 9	30 ± 17	0.465	0.41
Potassium (mg)	5029 ± 1471	4736 ± 1864	4956 ± 962	3816 ± 1281	0.011*	0.019*
Sodium (mg)	3831 ± 1209	3283 ± 966	3695 ± 969	2890.1 ± 827	0.144	0.028*
Zinc (mg)	21 ± 16	18 ± 9	21 ± 9	14 ± 7	0.068	0.109
Alcohol (g)	3.2 ± 4.6	4.0 ± 5.5	17 ± 17	6.7 ± 9.8	0.011*	0.409
Caffeine (mg)	155 ± 127	302 ± 157	186 ± 11	260 ± 178	0.715	0.007*

Data are presented as mean ± SD.

*indicates *p* < 0.05

## Discussion

The findings of the current research suggest that the restrictions put in place due to the current pandemic did not impact the training habits or sport-specific fitness levels of Masters-level cyclists in the short term (*c.* 4 months). However, the pandemic does appear to have impacted the intake of key nutrients such as alcohol, potassium, folate, vitamin A, omega-3 fatty acids and fiber, which may have detrimental effects on the health and fitness of this population in the long term.

No significant differences were observed in training volume or intensity in the population sampled. The results are supported by a lack of change in fitness measures such as time to exhaustion, heart rate and power output observed in the current research. These results are in contrast to findings of Lesser and Nienhuis (2020), who observed a decrease in physical activity levels in those who were already considered ‘inactive’ and an increase in activity in those considered ‘active’ in response to the COVID-19 pandemic in the general population. Similarly, both Ammar et al. (2020) and Moore et al. (2020) observed decreased levels of play and activity in children and adults, respectively, in the early stages of the pandemic compared to levels prior to the declaration of the pandemic. Therefore, the current population appears more resilient to changes in their environment compared to the general population. This could be due to the outdoor nature of cycling and the level of restriction in Canada where the sample was drawn from, as no restrictions were put in place for outdoor activities. In contrast, individuals from the general population or who participate in activities reliant on community infrastructure may have been affected to a greater degree due to facility closures (Ammar et al., 2020). Due to environmental considerations in Canada, many Canadian cyclists have the equipment and the skills required for indoor cycling in order to maintain their training schedule and fitness during the winter months. Such resources would allow this population to have minimal disturbances in their training habits even if restrictions on outdoor activities had been implemented. Further, many individuals began working from home, which may have increased the time available for cycling. Similarly, with many businesses and restaurants closed, roads may have been less occupied by vehicles, inviting more cyclists to take to the road.

Some differences were seen in performance measures of males and females, which is to be expected due to well-established sex differences in absolute strength and power output (Sandbakk et al., 2018). Females had greater average and maximal heart rate, despite no significant differences in age between males and females (*p* = 0.76). This is in agreement with Koenig and Thayer (2016), who report a higher average heart rate in females as well as Helgerud et al. (1990) who reported higher heart rates for a given running speed in females compared to performance-matched males.

The results of the current research suggest that the current pandemic did not impact the amount of time of our unique sample engaging in sedentary screen time. It is well accepted that sedentary behavior is an important, independent predictor of overall health (Hart et al., 2011). These findings are in contrast to that of Meyer et al. (2020), Romero-Blanco et al. (2020) and Moore et al. (2020) who observed increases in sedentary behavior in adults, Health Sciences university students and children, respectively, during the pandemic. Such contrasting results suggest that Masters cyclists have been incredibly resilient and have been able to maintain their fitness and training while not increasing sedentary time. A potential explanation for this could be that the pandemic struck at the beginning of Canadian spring, a time at which cyclists begin riding outdoors, which was not restricted due to the pandemic. It is of utmost importance that this population continues to minimize their time engaging in sedentary activities, as even marginal increases in sedentary behavior can have significant detrimental effects on cardiometabolic health (Hu et al., 2001).

The implication of more time spent at home may have various impacts on dietary intake. Increased time spent in the home environment may promote larger meal sizes and increased snack frequency and size, leading to a net increase in energy intake. Alternatively, decreased time spent outside of the home or to ‘eat out’ at restaurants may promote more structured meal patterns and increased prevalence of home meal preparation, as recommended by Canada’s Food Guide (www.food-guide.canada.ca) (Dai et al., 2020, Gallo et al., 2020). During the unprecedented time of the COVID-19 pandemic, researchers have commented that the virus will eventually subside, but potential negative health consequences due to decreased dietary quality and physical activity will result in increased cardiovascular disease risk beyond the COVID-19 virus (Mattioli et al., 2020). Recent reports of dietary habits during the pandemic have indicated increased energy intake in female university students (Gallo et al., 2020) and increased frequency of unhealthy food behaviors, such as binge eating and consumption of unhealthy foods as well as a decrease in binge drinking behavior (Ammar et al., 2020). The current research supports some of these findings, while contrasting others. There was no difference in energy intake in either males or females in response to the pandemic, which is different from the findings of Gallo et al. (2020). However, decreases in key nutrients such as fiber, omega-3 fatty acids, vitamin A, potassium and folate may represent a decrease in the nutritional density of the foods being consumed, which is similar to the findings of Ammar et al. (2020). These dietary changes may be detrimental to cardiovascular health of this population, as higher potassium levels are important in the prevention of hypertension (Ware et al., 2017) while fiber and omega-3 fatty acids are important in the prevention of cardiovascular disease (Hu et al., 2019; McRae et al., 2017), both of which increase the risk of implications related to COVID-19 (Nishiga et al., 2020; Ran et al., 2020). Similarly, an increase in alcohol intake found in the current study is in line with some research conducted throughout the pandemic in the general population (Chodkiewicz et al., 2020) but contrast Ammar et al. (2020) who found decreases in binge drinking, and a shift towards healthier habits, could be explained by their younger participants (18–35 years) and not being surrounded by other drinking peers. The current research also observed a slight increase in vitamin C intake (318 ± 247 mg v. 336 ± 343 mg) which, although statistically significant, is likely to have little clinical significance. The decreases in nutrient intake found in the current research may be due to recommendations for individuals to go to the grocery store only once per week. As such, their fruit and vegetable intake may have been limited to the shelf life of fresh produce or the underestimation of how much produce is required to last a full week. As fruits and vegetables are rich sources of vitamin A, potassium and fiber (Choi et al., 2015; Górska-Warsewicz et al., 2019), a reduced intake of these food groups might contribute to the current findings.

Various sex differences were observed in the present study with females consuming less energy, carbohydrate, fat, protein and various other nutrients. This is in line with the literature, which suggests that the increased energy intake by males also increases their capacity for macro- and micronutrient intake (Thomas et al., 2016). An increased energy intake in males is likely related to increased energy expenditure due to increased lean body mass (Sandbakk et al., 2018) and exercise energy expenditure, supported by the higher average and maximal power outputs by males observed in the current research, as well as due to an overall greater awareness of energy intake by females relative to their male peers (Heikura et al., 2018).

### Limitations

There are a number of limitations to our study, notably a small sample size given the online nature of our data collection. Due to social distancing guidelines, fitness testing took part in participants’ own homes; therefore, it is impossible to know whether participants truly went to their physical limits during both fitness tests or if they adhered to the request of completing the test under similar conditions. Further, our research was collected in May and June of 2020, participants, therefore, were asked to recall up to three months to report their ‘before COVID’ dietary intake, which could lead to reporting error.

## Conclusion

Despite the restrictions in place in order to slow the spread of the COVID-19 pandemic, Masters cyclists appear to be resilient in terms of their training habits, with no change in the quality or quantity of their training nor in their fitness levels. However, it would appear that the intake of some key nutrients has decreased while others increased, evidenced by no change in energy intake yet decreases in nutrients that are important for optimal health and performance. In addition, alcohol intake increased significantly during the pandemic, which may have long-term consequences on not only the training and fitness of this population but also their overall health. During the COVID-19 pandemic, Masters-level cyclists should continue to maintain their training habits and choose a diet rich in fruits, vegetables, whole grains and more plant-based proteins in order to improve their diet and that they practice moderation in regard to alcohol intake.

## References

[bibr1-02601060211002350] Ammar A Brach M Trabelsi K, et al. (2020) Effects of COVID-19 home confinement on eating behaviour and physical activity: Results of the ECLB-COVID19 International Online Survey. Nutrients 12(6): 1583. 32481594 10.3390/nu12061583 PMC7352706

[bibr2-02601060211002350] Appleby KM Dieffenbach K (2016) ‘Older and faster’: Exploring elite masters cyclists’ involvement in competitive sport. The Sport Psychologist 30(1): 13–23.

[bibr3-02601060211002350] Bai Y Yao L Wei T, et al. (2020) Presumed asymptomatic carrier transmission of COVID-19. JAMA 323(14): 1406–1407. 32083643 10.1001/jama.2020.2565 PMC7042844

[bibr4-02601060211002350] Baker J Horton S Weir P (eds) (2009) The Masters Athlete: Understanding the role of sport and exercise in optimizing aging. Abingdon: Routledge.

[bibr5-02601060211002350] Bouillod A Pinot J Soto-Romero G, et al. (2017) Validity, sensitivity, reproducibility, and robustness of the PowerTap, Stages, and Garmin Vector power meters in comparison with the SRM device. International Journal of Sports Physiology and Performance 12(8): 1023–1030. 27967278 10.1123/ijspp.2016-0436

[bibr6-02601060211002350] Bradley K Kolt GS Williams MM (2005) Motives for participating in international level masters athletics. New Zealand Journal of Sports Medicine 3(1): 4–12.

[bibr7-02601060211002350] Butler MJ Barrientos RM (2020) The impact of nutrition on COVID-19 susceptibility and long-term consequences. Brain, Behavior, and Immunity 87: 53–54. 32311498 10.1016/j.bbi.2020.04.040 PMC7165103

[bibr8-02601060211002350] Cavanagh P Evans J Fiatarone M, et al. (1998) Exercise and physical activity for older adults. Medicine and Science in Sports and Exercise 30(6): 1–29. 9624662

[bibr9-02601060211002350] CDC COVID-19 Response Team (2020) Severe outcomes among patients with coronavirus disease 2019 (COVID-19) – United States, February 12–March 16, 2020. Morbidity and Mortality Weekly Report 69(12): 343–346. 32214079 10.15585/mmwr.mm6912e2 PMC7725513

[bibr10-02601060211002350] Chodkiewicz J Talarowska M Miniszewska J, et al. (2020) Alcohol consumption reported during the COVID-19 pandemic: The initial stage. International Journal of Environmental Research and Public Health 17(13): 4677. 32610613 10.3390/ijerph17134677 PMC7369979

[bibr11-02601060211002350] Choi Y Lee JE Bae JM, et al. (2015) Vegetable intake, but not fruit intake, is associated with a reduction in the risk of cancer incidence and mortality in middle-aged Korean men. The Journal of Nutrition 145(6): 1249–1255. 25878208 10.3945/jn.114.209437

[bibr12-02601060211002350] Concannon LG Grierson MJ Harrast MA (2012) Exercise in the older adult: From the sedentary elderly to the masters athlete. Physical Medicine and Rehabilitation 4(11): 833–839. 10.1016/j.pmrj.2012.08.007 23174546

[bibr13-02601060211002350] Csizmadi I Kahle L Ullman R, et al. (2007) Adaptation and evaluation of the National Cancer Institute’s Diet History Questionnaire and nutrient database for Canadian populations. Public Health Nutrition 10(1): 88–96. 17212847 10.1017/S1368980007184287

[bibr14-02601060211002350] Dai Z Kroeger CM Lawrence M, et al. (2020) Comparison of methodological quality between the 2007 and 2019 Canadian dietary guidelines. Public Health Nutrition 23: 2879–2885. 32552917 10.1017/S1368980020000956 PMC10200617

[bibr15-02601060211002350] Gallo LA Gallo TF Young SL, et al. (2020) The impact of isolation measures due to COVID-19 on energy intake and physical activity levels in Australian university students. Nutrients 12: 1865. 32585830 10.3390/nu12061865 PMC7353248

[bibr16-02601060211002350] Greenstone M Nigam V (2020) Does social distancing matter? University of Chicago, Becker Friedman Institute for Economics Working Paper No. 2020–26.

[bibr17-02601060211002350] Górska-Warsewicz H Rejman K Laskowski W, et al. (2019) Food sources of potassium in the average Polish diet. Nutrients 11(12): 2905. 31805745 10.3390/nu11122905 PMC6950722

[bibr18-02601060211002350] Hart TL Swartz AM Cashin SE, et al. (2011) How many days of monitoring predict physical activity and sedentary behaviour in older adults? International Journal of Behavioral Nutrition and Physical Activity 8(1): 62. 21679426 10.1186/1479-5868-8-62 PMC3130631

[bibr19-02601060211002350] Heikura IA Stellingwerff T Burke LM (2018) Self-reported periodization of nutrition in elite female and male runners and race walkers. Frontiers in Physiology 9: 1732. 30559680 10.3389/fphys.2018.01732 PMC6286987

[bibr20-02601060211002350] Helgerud J Ingjer F Strømme SB (1990) Sex differences in performance-matched marathon runners. European Journal of Applied Physiology and Occupational Physiology 61(5–6): 433–439. 2079063 10.1007/BF00236064

[bibr21-02601060211002350] Hu FB Leitzmann MF Stampfer MJ, et al. (2001) Physical activity and television watching in relation to risk for type 2 diabetes mellitus in men. Archives of Internal Medicine 161(12): 1542–1548. 11427103 10.1001/archinte.161.12.1542

[bibr22-02601060211002350] Hu Y Hu FB Manson JE (2019) Marine omega-3 supplementation and cardiovascular disease: An updated meta-analysis of 13 randomized controlled trials involving 127 477 participants. Journal of the American Heart Association 8(19): e013543. 31567003 10.1161/JAHA.119.013543 PMC6806028

[bibr23-02601060211002350] Jiménez-Pavón D Carbonell-Baeza A Lavie CJ (2020) Physical exercise as therapy to fight against the mental and physical consequences of COVID-19 quarantine: Special focus in older people. Progress in Cardiovascular Diseases 63: 386–388. 32220590 10.1016/j.pcad.2020.03.009 PMC7118448

[bibr24-02601060211002350] Koenig J Thayer JF (2016) Sex differences in healthy human heart rate variability: A meta-analysis. Neuroscience & Biobehavioral Reviews 64: 288–310. 26964804 10.1016/j.neubiorev.2016.03.007

[bibr25-02601060211002350] Lange KW Nakamura Y (2020) Movement and nutrition in COVID-19. Movement and Nutrition in Health and Disease 4: 89–94.

[bibr26-02601060211002350] Lau H Khosrawipour V Kocbach P, et al. (2020) The positive impact of lockdown in Wuhan on containing the COVID-19 outbreak in China. Journal of Travel Medicine 27(3): taaa037. 10.1093/jtm/taaa037 PMC7184469 32181488

[bibr27-02601060211002350] Lesser IA Nienhuis CP (2020) The impact of COVID-19 on physical activity behavior and well-being of Canadians. International Journal of Environmental Research and Public Health 17(11): 3899. 32486380 10.3390/ijerph17113899 PMC7312579

[bibr28-02601060211002350] Madsen K Pedersen PK Djurhuus MS, et al. (1993) Effects of detraining on endurance capacity and metabolic changes during prolonged exhaustive exercise. Journal of Applied Physiology 75(4): 1444–1451. 8282588 10.1152/jappl.1993.75.4.1444

[bibr29-02601060211002350] Mattioli AV Sciomer S Cocchi C, et al. (2020) Quarantine during COVID-19 outbreak: Changes in diet and physical activity increase the risk of cardiovascular disease. Nutrition, Metabolism and Cardiovascular Diseases 30: 1409–1417. 10.1016/j.numecd.2020.05.020 PMC7260516 32571612

[bibr30-02601060211002350] Maula A LaFond N Orton E, et al. (2019) Use it or lose it: A qualitative study of the maintenance of physical activity in older adults. BMC Geriatrics 19(1): 349. 31830900 10.1186/s12877-019-1366-x PMC6909612

[bibr31-02601060211002350] McRae MP (2017) Dietary fiber is beneficial for the prevention of cardiovascular disease: An umbrella review of meta-analyses. Journal of Chiropractic Medicine 16(4): 289–299. 29276461 10.1016/j.jcm.2017.05.005 PMC5731843

[bibr32-02601060211002350] Meyer J McDowell C Lansing J, et al. (2020) Changes in physical activity and sedentary behaviour due to the COVID-19 outbreak and associations with mental health in 3,052 US adults. Cambridge Open Engage. Cambridge: Cambridge Open Engage.

[bibr33-02601060211002350] Moore SA Faulkner G Rhodes RE, et al. (2020) Impact of the COVID-19 virus outbreak on movement and play behaviours of Canadian children and youth: A national survey. International Journal of Behavioral Nutrition and Physical Activity 17(1): 1–11. 32631350 10.1186/s12966-020-00987-8 PMC7336091

[bibr34-02601060211002350] Nishiga M Wang DW Han Y, et al. (2020) COVID-19 and cardiovascular disease: From basic mechanisms to clinical perspectives. Nature Reviews Cardiology 17: 543–558. 32690910 10.1038/s41569-020-0413-9 PMC7370876

[bibr35-02601060211002350] Priego Quesada JI Kerr ZY Bertucci WM, et al. (2018) The categorization of amateur cyclists as research participants: Findings from an observational study. Journal of Sports Sciences 36(17): 2018–2024. 29369014 10.1080/02640414.2018.1432239

[bibr36-02601060211002350] Ran J Song Y Zhuang Z, et al. (2020) Blood pressure control and adverse outcomes of COVID-19 infection in patients with concomitant hypertension in Wuhan, *China.* Hypertension Research 43: 1267–1276. 32855527 10.1038/s41440-020-00541-w PMC7450040

[bibr37-02601060211002350] Romero-Blanco C Rodríguez-Almagro J Onieva-Zafra MD, et al. (2020) Physical activity and sedentary lifestyle in university students: Changes during confinement due to the Covid-19 pandemic. International Journal of Environmental Research and Public Health 17(18): 6567. 32916972 10.3390/ijerph17186567 PMC7558021

[bibr38-02601060211002350] Sandbakk Ø Solli GS Holmberg HC (2018) Sex differences in world-record performance: The influence of sport discipline and competition duration. International Journal of Sports Physiology and Performance 13(1): 2–8. 28488921 10.1123/ijspp.2017-0196

[bibr39-02601060211002350] Terbizan DJ Dolezal BA Albano C (2002) Validity of seven commercially available heart rate monitors. Measurement in Physical Education and Exercise Science 6(4): 243–247.

[bibr40-02601060211002350] Thomas DT Erdman KA Burke LM (2016) Position of the Academy of Nutrition and Dietetics, Dietitians of Canada, and the American College of Sports Medicine: Nutrition and athletic performance. Journal of the Academy of Nutrition and Dietetics 116(3): 501–528. 26920240 10.1016/j.jand.2015.12.006

[bibr41-02601060211002350] van Erp T Foster C de Koning JJ (2019) Relationship between various training-load measures in elite cyclists during training, road races, and time trials. International Journal of Sports Physiology and Performance 14(4): 493–500. 30300025 10.1123/ijspp.2017-0722

[bibr42-02601060211002350] Ware LJ Charlton K Schutte AE, et al. (2017) Associations between dietary salt, potassium and blood pressure in South African adults: WHO SAGE Wave 2 Salt & Tobacco. Nutrition, Metabolism and Cardiovascular Diseases 27(9): 784–791. 10.1016/j.numecd.2017.06.017 28800936

[bibr43-02601060211002350] Wintergerst ES Maggini S Hornig DH (2007) Contribution of selected vitamins and trace elements to immune function. Annals of Nutrition and Metabolism 51(4): 301–323. 17726308 10.1159/000107673

